# Effect of dentin pre-treatment with Bioglass F18 suspensions on surface characteristics and bond strength of endodontic sealers

**DOI:** 10.1007/s00784-026-07022-3

**Published:** 2026-07-24

**Authors:** Marcela de Come Ramos, Pedro Henrique Fiorin de Souza, Fernanda Ferrari Esteves Torres, Marina Trevelin Souza, Edgar Dutra Zanotto, Airton Oliveira Santos-Junior, Juliane Maria Guerreiro-Tanomaru, Mario Tanomaru-Filho

**Affiliations:** 1 Department of Restorative Dentistry, School of Dentistry, São Paulo State University (UNESP), Rua Humaitá, 1680, Araraquara, SP CEP 14801-903 Brazil; 2 Department of Materials Engineering, Vitreous Materials Laboratory (LaMaV), Federal University of São Carlos (UFSCar), São Carlos, SP Brazil

**Keywords:** Bioactive glass, Biomineralization, Bond strength, Dental materials, Microscopy electron scanning, Mechanical tests, Root canal sealer

## Abstract

**Objectives:**

To investigate whether dentin pre-treatment with bioactive glass F18 suspensions modifies dentin surface characteristics and influences the bond strength of bioceramic and epoxy resin-based endodontic sealers.

**Material and methods:**

Extracted human root canals were treated with distilled water, PBS or Bioglass F18 suspensions (2.5%, 5%, or 10%). Dentin surface morphology and elemental composition were analyzed by scanning electron microscopy and energy-dispersive X-ray spectroscopy. Bovine dentin specimens were treated with distilled water or 5% F18 and filled with a bioceramic (Bio-C Sealer) or epoxy resin–based sealer (AH Plus) for interfacial and mechanical analyses. The material–dentin interface was evaluated by micro–Fourier transform infrared spectroscopy after PBS immersion. Push-out and tensile bond strength tests were performed. Statistical analysis was performed using appropriate parametric or non-parametric tests according to data distribution. The level of significance was set at α = 0.05.

**Results:**

Bioglass F18 promoted the deposition of bioactive material on the dentin surface, with comparable effects observed at 5% and 10% concentrations. All F18 concentrations significantly increased the bond strength of both sealers compared with controls (*p* < 0.05). In tensile testing, dentin pre-treatment with 5% F18 resulted in significantly higher bond strength (*p* < 0.05). AH Plus exhibited higher bond strength than Bio-C Sealer in both mechanical tests (*p* < 0.05).

**Conclusions:**

Dentin pre-treatment with Bioglass F18 enhanced dentin bioactivity and improved the sealer–dentin adhesion for both Bio-C Sealer and AH Plus. The effects were concentration-dependent across different outcomes, with 5% F18 showing favorable performance in tensile bond strength and comparable behavior to 10% F18 in push-out testing.

**Clinical relevance:**

Dentin pre-treatment with Bioglass F18 may improve sealer–dentin interfacial performance under in vitro conditions and could represent a potential adjunctive approach in root canal sealing strategies, pending further long-term and in vivo investigations.

## Introduction

Root canal filling aims to achieve a three-dimensional seal capable of preventing microbial leakage and endodontic reinfection [1–3]. The long-term success of endodontic treatment is closely associated with the integrity of the dentin–sealer interface, since inadequate adaptation and void formation may compromise sealing effectiveness [4, 5]. In this context, bioceramic sealers have gained increasing attention due to their calcium silicate–based composition, biocompatibility, and bioactive properties [6–11]. These materials can induce the formation of an apatite-like calcium phosphate layer at the sealer–dentin interface [12, 13], which may promote tag-like structures and enhance micromechanical interlocking between the sealer and dentin.

Within this scenario, F18 bioactive glass (F18; Vetra Biomaterials Ltda., Ribeirão Preto, SP, Brazil), developed by the Vitreous Materials Laboratory (LaMaV), Department of Materials Engineering, Federal University of São Carlos (UFSCar, São Carlos, SP, Brazil), is a bioactive glass biomaterial composed of silicon dioxide, sodium oxide, potassium oxide, magnesium oxide, calcium oxide, and phosphorus pentoxide [12]. This material exhibits bioactive potential, antimicrobial and antibiofilm activity, as well as biocompatibility [13–19]. In addition, F18 has been shown to induce osteoblast and fibroblast proliferation [12, 14, 17, 18], thereby promoting tissue repair [13], in addition to demonstrating the ability to promote bone new formation [20]. Its bioactivity can be evidenced by the formation of a hydroxycarbonate apatite layer on the dentin surface [13, 17, 18]. Moreover, final irrigation with F18 in regenerative endodontic procedures in rats promoted cell proliferation and increased osteocalcin expression, a marker that plays an important role in mineralized tissue formation [21]. Furthermore, F18 bioactive glass has been shown to promote surface mineralization of gutta-percha and improve its bond strength [22]. Despite these promising biological properties, the potential of F18 suspensions on the dentin surface and their interaction with endodontic sealers remain unexplored. The bioactive potential of F18 may therefore be beneficial for the dentin–sealer interface, enhancing mechanical retention and chemical interaction.

The use of ready-to-use bioceramic root canal sealers has increased in recent years due to their clinical applicability and favorable biological properties, including bioactivity [6, 23]. The ready-to-use bioceramic Bio-C Sealer (BCS; Angelus, Londrina, PR, Brazil) exhibits biocompatibility, calcium ion release, alkaline pH, and favorable physicochemical properties, such as adequate flow and radiopacity [24–27]. Although contemporary bioceramic sealers have advantages, further improvements can optimize their clinical performance [28]. BCS exhibits bioactivity, but has lower bond strength to dentin when compared to AH Plus epoxy resin-based cement sealer. (AHP; Dentsply DeTrey, Konstanz, Germany) [16, 29, 30]. AHP is widely regarded as the gold standard for comparisons with other sealers due to its excellent physicochemical properties and consistently high bond strength [31–33]. Its adhesion to root canal walls is attributed to both chemical and mechanical interactions with dentin, including chemical interactions with dentin components and micromechanical interlocking through penetration into dentinal irregularities [34, 35], which contribute to its superior bond strength [16, 29, 36, 37]. Despite these favorable adhesive properties, AHP lacks bioactivity and has been associated with a more pronounced inflammatory response than bioceramic sealers, particularly at longer evaluation periods [11, 38].

The bioactive potential of calcium silicate-based sealers is evidenced by the deposition of apatite-like calcium phosphate along the sealer-dentin interfacial layer [12], but their bond strength to root canal dentin needs to be improved. On the other hand, epoxy resin-based sealers, such as AH Plus, despite demonstrating better mechanical performance, exhibit less favorable biological responses and limited bioactivity [11]. Therefore, strategies that enhance dentin biomineralization before root canal obturation may represent a promising approach to improve the quality of the dentin-sealer interface, both for bioceramic and resin-based sealers. In this context, bioactive material suspensions represent an adjuvant approach to increase the reactivity of the dentin surface and promote early mineral deposition, thus reinforcing the interfacial layer and improving mechanical and chemical retention. Although bioactive glass materials have been consistently associated with apatite formation on dentin surfaces, their application as a dentin pretreatment needs to be further explored [39].

Accordingly, this study investigated whether pre-treatment of root canal dentin with an experimental F18 bioactive glass suspension alters dentin surface features, promotes surface biomineralization, and enhances interfacial quality and bond strength of bioceramic- and resin-based endodontic sealers. This study aimed to provide a comprehensive evaluation of the bioactive potential of F18 and its impact on dentin–sealer interaction by integrating morphological, chemical, and mechanical analyses. The null hypothesis stated that dentin pre-treatment with F18 would not produce significant changes in dentin surface properties, interfacial chemical interactions, or sealer–dentin bond strength.

## Materials and methods

**Fig. 1:**
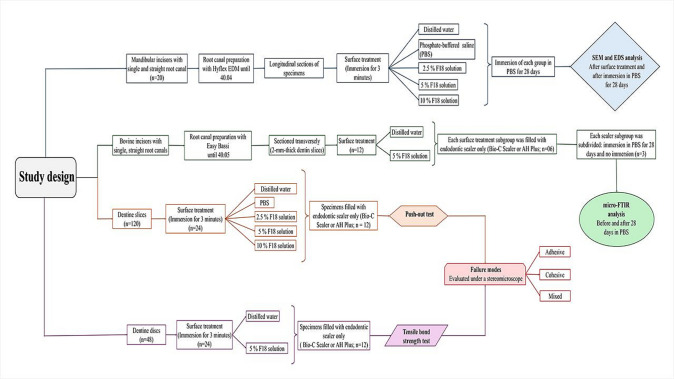
Flowchart summarizing the experimental design and procedural sequence of the study, including dentin surface treatments, qualitative (SEM/EDS) and interfacial chemical (micro-FTIR) analyses, mechanical bond strength tests (push-out and tensile), and failure mode evaluation

The materials used, their manufacturers, chemical composition, and proportions are summarized in Table 1. Bioglass F18 was based on a previously characterized formulation. F18 bioactive glass was synthesized by mixing the precursor oxides, followed by melting at 1450 °C in a platinum crucible for 3 h. After melting, the material was rapidly quenched in water to obtain a glass frit, which was subsequently dried at 100 °C for 24 h**.** The dried material was then ground in a planetary mill (Pulverisette; Fritsch, Germany) until a mean particle size of approximately 5 µm was achieved [40, 41]. The F18 glass powder was weighed and mixed with distilled and deionized water to obtain final concentrations of 2.5%, 5%, and 10% (w/w). The suspensions were homogenized using a vortex mixer immediately before use to ensure adequate dispersion of the particles. In this study, to receive the treatment, the samples were immersed in 30 mL of the F18 suspension for 3 min. The overall experimental workflow and procedural sequence of the study are illustrated in Fig. 1.

**Table 1:** Materials, manufacturers, composition, and presentation/experimental concentrations

Materials	Manufacturers	Composition	Presentation/Concentration
Bioglass F18 (F18)	Vetra Biomaterials Ltda., Ribeirão Preto, SP, Brazil; developed by the Vitreous Materials Laboratory (LaMaV), Department of Materials Engineering, Federal University of São Carlos (UFSCar), São Carlos, SP, Brazil	Precursors used for synthesis: Na₂CO₃, K₂CO₃, K₂HPO₄, Na₂HPO₄, CaCO₃, SiO₂, Al₂O₃, ZnO, SrCO₃ (wt%); microparticles (D50 = 5 µm)	2.5%, 5% and 10% suspensions
Bio-C Sealer (BCS)	Angelus (Londrina, PR, Brazil)	Calcium silicates, calcium aluminate, calcium oxide, zirconium oxide, iron oxide, silicon dioxide and dispersing agent	Ready-to-use
AH Plus (AHP)	Dentsply DeTrey (Konstanz, Germany)	Paste A: bisphenol-A and bisphenol-F epoxy resin, CaWO₄, ZrO₂, Fe₂O₃, and silica; Paste B: dibenzyldiamine, aminoadamantane, CaWO₄, ZrO₂, silica, and silicone	Paste-paste (1 g:1 g)

F18, Bioglass F18; BCS, Bio-C Sealer; AHP, AH Plus

Na₂CO₃, sodium carbonate; K₂CO₃, potassium carbonate; K₂HPO₄, dipotassium phosphate; Na₂HPO₄, disodium phosphate; CaCO₃, calcium carbonate; SiO₂, silicon dioxide; Al₂O₃, aluminum oxide; ZnO, zinc oxide; SrCO₃, strontium carbonate; CaWO₄, calcium tungstate; ZrO₂, zirconium oxide; Fe₂O₃, iron(III) oxide

### Ethical approval

This study was approved by the local Research Ethics Committee (protocol number: 67998123.2.0000.5416). All procedures involving human specimens were conducted in accordance with the principles of the World Medical Association Declaration of Helsinki.

### Sample size determination and specimen selection

Sample size calculation was performed using G*Power 3.1 software (Universität Düsseldorf, Germany). Based on a statistical power of 95% (1–β = 0.95), a significance level of 5% (α = 0.05), and an effect size estimate of 1.7 derived from previously reported mean differences and standard deviations in studies with comparable methodologies evaluating push-out bond strength [42, 43], a minimum of 12 specimens per group was established for the quantitative mechanical analyses performed in this study. The effect size estimate was obtained from studies with comparable experimental designs and outcome variables, including Karobari et al. [42] and Lucas et al. [43]. This estimate was used as a reference for study planning based on previously published bond strength studies with comparable methodologies and was not intended to represent any specific comparison among the experimental groups evaluated in the present investigation.

Accordingly, sixty bovine incisors with straight root canals and fully formed apices were selected after radiographic examination using a digital radiography system (RVG 6100; Kodak Dental Systems, NY, USA). For qualitative surface and compositional analyses, twenty extracted human mandibular incisors with a single, straight root canal were selected for scanning electron microscopy (SEM) and energy-dispersive X-ray spectroscopy (EDS) analyses [44, 45].

Human teeth were used for SEM and EDS analyses to allow a clinically relevant assessment of dentin surface morphology and mineral deposition patterns, as their tubular architecture more closely reflects in vivo conditions. In contrast, bovine teeth were selected for Micro–Fourier Transform Infrared Spectroscopy (micro-FTIR) and mechanical testing due to their greater availability, dimensional uniformity, and suitability for specimen standardization, which enhances reproducibility and reduces inter-sample variability in bond strength evaluation [46–49].

The dentin specimens were standardized through controlled root length, root canal preparation, and slice thickness, combined with cylindrical preparations and random allocation to minimize the impact of anatomical variability.

### SEM analysis of dentin treatment in human teeth

Initial selection was based on periapical radiographs obtained in buccolingual and mesiodistal projections to confirm a single, straight canal with a fully formed apex and no evidence of internal resorption or prior endodontic treatment. The teeth were then evaluated under a stereomicroscope (10 ×) (Leica Microsystems-M80, Wetzlar, Germany) to exclude specimens with cracks, fractures, or external root defects. Root canal preparation was performed 1 mm short of the apical foramen using HyFlex EDM rotary instruments (25/0.12, 25/0.08, and 40/0.04; Coltene, Switzerland), operated at 500 revolutions per minute (rpm) and 2 N·cm torque. During instrumentation, irrigation was performed with 5 mL of 2.5% sodium hypochlorite (NaOCl) delivered using an Ultradent syringe (South Jordan, UT, USA) and a 30-gauge Navitip needle (Ultradent Products). Final root canal cleaning consisted of sequential irrigation with 5 mL of 2.5% NaOCl, 3 mL of 17% ethylenediaminetetraacetic acid (EDTA) (Biodinâmica, Ibiporã, PR, Brazil), and 5 mL of distilled water. Each irrigant was activated using an Easy Clean instrument (Easy Bassi, Belo Horizonte, MG, Brazil) in low-speed rotary motion, following a standardized protocol consisting of three cycles, each lasting 15 s.

The specimens were longitudinally sectioned using carborundum discs (Dentorium, New York, United States of America) coupled to a low-speed handpiece (KaVo, Biberach, Germany). The sections were performed approximately 1 mm from the root canal to allow separation of the halves using a chisel. After sectioning, the specimens were subjected to an additional cleaning protocol consisting of immersion in 2.5% sodium hypochlorite, 17% EDTA, and distilled water, in order to remove debris and smear residues generated during the cutting procedure. This step aimed to standardize the dentin surface and prevent potential interference with the subsequent analyses. The specimens were then allocated according to the surface treatment suspensions: distilled water, phosphate-buffered saline (PBS), 2.5% F18 suspension, 5% F18 suspension, or 10% F18 suspension. The specimens were randomly assigned to the experimental groups by manual allocation. To reduce potential selection bias related to specimen origin, care was taken to restrict the inclusion of specimens from the same tooth to a single experimental group, thereby maintaining independence among group. The tested suspensions were applied for 3 min using a standardized volume across all experimental groups. This protocol was selected to simulate a final irrigation protocol after root canal preparation, ensuring methodological standardization between dentin and the tested suspensions under controlled laboratory conditions.

Qualitative evaluation of dentinal tubule obliteration was performed by SEM (JEOL JSM-6610LV, Tokyo, Japan). Four samples from each group were analyzed immediately after surface treatment, while the other four samples were analyzed after immersion in PBS for 28 days, allowing for a comparison between samples that were and were not immersed in PBS. Specimens allocated to the PBS condition were individually immersed in 5 mL of PBS for 28 days at 37 °C. The PBS solution was not replaced during this period. This static immersion protocol was used to evaluate ion exchange and mineral deposition under controlled confined conditions.

SEM images were obtained at magnifications of 200 ×, 300 ×, 500 ×, and 700 ×. All SEM images were evaluated by an examiner with over 20 years of experience (J.M.G.T), who was blinded to the experimental groups. For the analysis, two areas from each third (cervical, middle, and apical) were evaluated, resulting in a total of 6 regions analyzed per specimen.

SEM and EDS data were subjected to purely descriptive and qualitative assessments; thus, inferential statistical analyses were not applied. Representative micrographs were selected from the evaluated regions to illustrate the predominant morphological characteristics of each experimental group. The same dentin regions were not tracked before and after PBS immersion; therefore, the analyses were based on representative fields from different specimens within each experimental condition.

Dentinal tubule obliteration was classified using a six-point scoring system:

- **Score 0** – dentin surface with open dentinal tubules;
- **Score 1** – dentin surface with partially open tubules (≥ 50%);
- **Score 2** – partial coverage of the dentin surface by F18, with no open tubules;
- **Score 3** – partial coverage of the dentin surface by bioactive material, with no open tubules;
- **Score 4** – complete coverage of the dentin surface by F18, with no open tubules;
- **Score 5** – complete coverage of the dentin surface by bioactive material, with no open tubules.

### EDS analysis for bioactive potential

EDS spectra were acquired and analyzed following standardized protocols using a SEM equipped with an EDS detector (JEOL JSM-6610LV, Tokyo, Japan). The analysis was performed to assess the elemental composition of dentin surfaces after the different surface treatments and after immersion in PBS for 28 days, with emphasis on calcium- and phosphorus-related signals indicative of biomineralization.

### micro-FTIR analysis

For the selection of bovine teeth, digital radiographic examinations were performed to confirm the presence of a single, straight root canal. After selection, the crowns were removed at the cementoenamel junction using carborundum discs coupled to a low-speed motor and the roots were standardized to a final length of 25 mm. Root canal patency was confirmed using a #10 K-file (Dentsply Sirona, Switzerland), and the working length was established 1 mm short of the apical foramen. Root canal preparation was performed using an Orifice Shaper #15.10, followed by #25.05, #30.05, and #40.05 rotary instruments (Easy Bassi, Belo Horizonte, MG, Brazil), operated at 500 rpm and 2 N·cm torque. Irrigation during preparation was performed with 5 mL of 2.5% NaOCl. Final root canal cleaning consisted of sequential irrigation with 5 mL of 2.5% NaOCl, 3 mL of 17% EDTA, and 5 mL of distilled water. Each irrigant was activated using an Easy Clean instrument (Easy Bassi, Belo Horizonte, MG, Brazil) using low-speed rotary motion, following a standardized protocol of three cycles of 15 s.

The roots were then sectioned transversely using a precision cutting machine (Isomet 1000; Buehler Ltd., Lake Bluff, IL, USA) to obtain 2-mm-thick dentin slices. The specimens were allocated into a control group treated with distilled water (*n =* 12) and an experimental group treated with 5% F18 (*n =* 12) and further subdivided according to the endodontic sealer used (*n =* 6 per subgroup): Bio-C Sealer or AH Plus. Bio-C Sealer was applied using the manufacturer’s syringe and plastic delivery tips. AH Plus was prepared by mixing equal lengths of pastes A and B for 30 s using a #24 metal spatula (Golgran, Brazil). Before sealer placement, the root canal spaces were dried using absorbent paper points. The same drying protocol was applied to all specimens to standardize dentin surface conditions prior to sealer insertion.

After sealer placement, the specimens were stored at 37 °C and 100% relative humidity for 3 days. Subsequently, three specimens from each subgroup were immersed in PBS for 28 days, while the remaining samples were maintained as non-immersed controls. After PBS storage, the immersed specimens were kept in a desiccator for 3 days prior to analysis.

Interfacial chemical analyses were performed using a Fourier-transform infrared spectrophotometer (VERTEX 70; Bruker, Billerica, MA, USA) coupled to a Hyperion 200 infrared microscope (Bruker). The specimens were fixed on the microscope stage, and 4 × and 15 × objectives were used to locate the regions of interest. For each specimen, spectra were acquired from three standardized points: dentin, the dentin–sealer interface, and the sealer. Spectral acquisition was performed in the 4000–600 cm⁻^1^ range, with a resolution of 4 cm⁻^1^ and 128 scans per spectrum. Representative images of the analyzed regions were captured to document the measurement sites.

### Push-out bond strength

Bovine tooth roots were sectioned into 2-mm-thick slices using a precision cutting machine (Isomet 1000; Buehler). Root canals were prepared using a standardized protocol with a dental surveyor and a 702-conical trunk carbide drill (JET Carbide Burs, Kerr Dental, Canada) to obtain cylindrical preparations and ensure standardized penetration in both cervico–apical and apico–cervical directions [27]. The final canal diameter was standardized at approximately 1.5 mm.

The specimens were immersed in 30 mL of 2.5% NaOCl, followed by immersion in 30 mL of 17% EDTA for 3 min. Subsequently, the samples were immersed in 30 mL of the respective surface treatment suspensions for 3 min: distilled water, PBS, 2.5% F18, 5% F18, or 10% F18. The specimens were then subdivided (*n =* 12) and filled with either Bio-C Sealer, using the manufacturer’s delivery system, or AH Plus, using standardized condensers. Specimens were filled with sealer only, without gutta-percha, to allow direct evaluation of the sealer–dentin interface. All specimens were stored at 37 °C under relative humidity for 7 days to allow complete setting of the sealers.

Push-out testing was performed using a universal testing machine operating with a 1-kN load cell at a constant crosshead speed of 0.5 mm/min until material displacement occurred [27]. A stainless-steel cylindrical punch with a diameter of 1.3 mm was used. The maximum load at failure was recorded in Newtons (N) and converted to Megapascals (MPa) according to the formula:

$$\mathrm{B}\mathrm{o}\mathrm{n}\mathrm{d}\;\mathrm{s}\mathrm{t}\mathrm{r}\mathrm{e}\mathrm{n}\mathrm{g}\mathrm{t}\mathrm{h}\;(\mathrm{M}\mathrm{P}\mathrm{a})\;=\;\mathrm{N}\;/\;\mathrm{A},$$

where *N* represents the maximum load and *A* the bonded surface area (mm^2^).

The bonded surface area was calculated using the formula A = 2πrh, where π = 3.14, *r* is the standardized root canal radius, and *h* is the thickness of each specimen (mm), measured using a digital caliper.

### Tensile bond strength

**Fig. 2:**
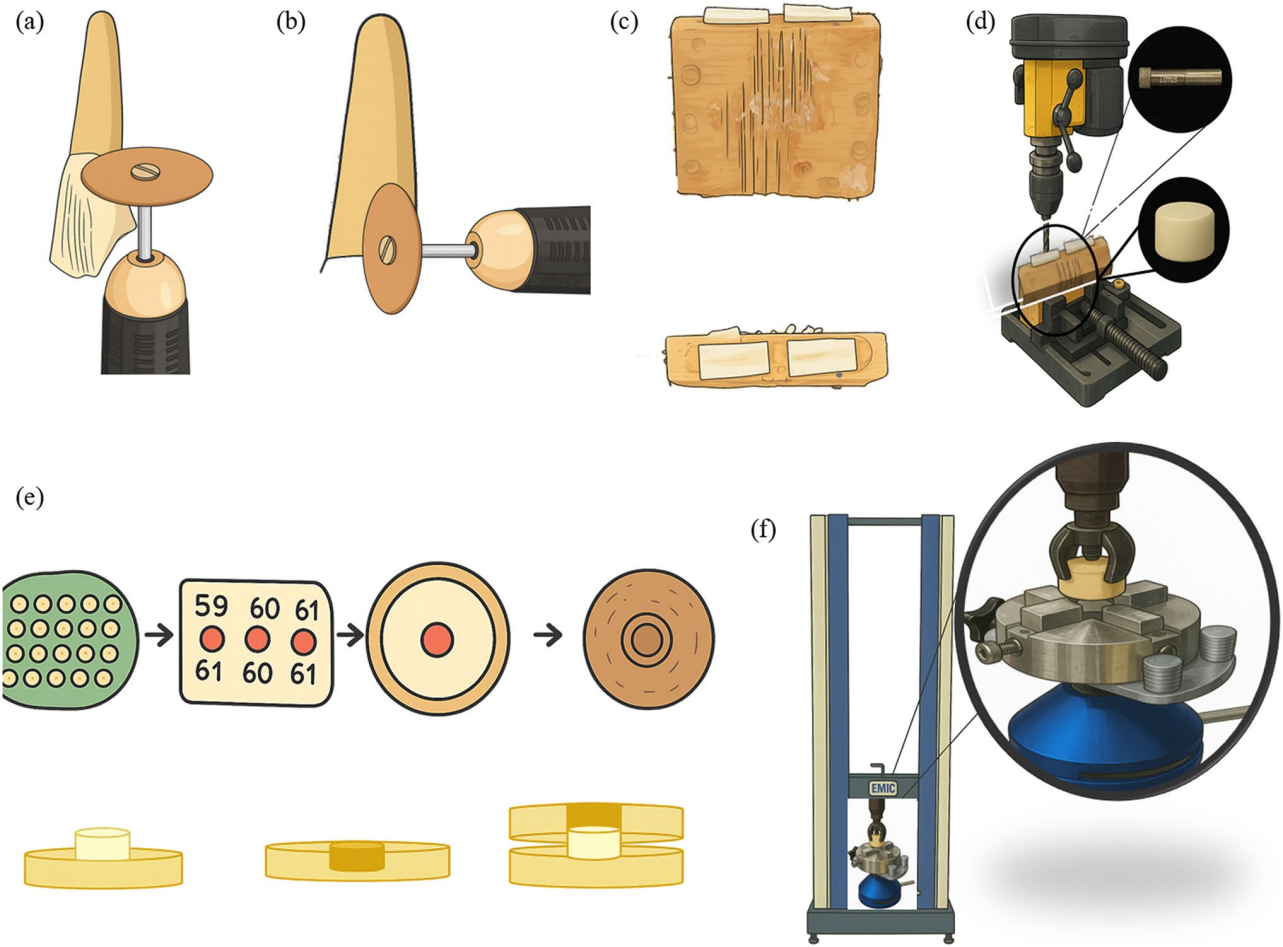
Schematic representation of specimen preparation and tensile bond strength testing. **a** Selection and sectioning of bovine tooth crowns. **b** Transverse sectioning of the roots. **c** Positioning of the roots in wooden fixtures. **d** Preparation of dentin discs using a bench-top drill coupled with a diamond trephine bur. **e** Sequence of dentin disc molding, wax pattern fabrication, and embedding in autopolymerizing acrylic resin to obtain a standardized fit between dentin and sealer specimens. **f** Schematic illustration of the tensile bond strength test, highlighting the custom-designed tensile device developed by the authors

Root dentin blocks were obtained from bovine incisor roots sectioned longitudinally using a precision cutting machine. Dentin discs measuring 4.5 mm in diameter and 2 mm in height were prepared using a bench-top drill press equipped with an 8-mm outer-diameter diamond trephine bur. A standardized system was developed to ensure consistent contact between the dentin specimens and the endodontic sealer (Fig. 2). Each dentin disc was positioned under a mold designed to create a standardized space for sealer placement.

Dentin surface treatment was performed by immersion in 30 mL of 2.5% NaOCl for 3 min, followed by treatment with 17% EDTA and distilled water. Final surface treatment was then performed with either distilled water or 5% F18, with the latter concentration selected based on the SEM and EDS findings, which demonstrated more homogeneous and stable surface mineral deposition compared with 2.5% F18, while no additional qualitative benefits were observed with 10% F18. After final surface treatment with distilled water or 5% F18, the specimens were not rinsed. This procedure was adopted to preserve the bioactive particles deposited on the dentin surface. Excess surface moisture was gently removed with absorbent paper for approximately 10 s, without air-drying or complete dehydration of the dentin surface, before sealer placement. The specimens were subsequently subdivided (*n =* 12) and filled with either Bio-C Sealer or AH Plus. Specimens were filled with sealer only, without gutta-percha, to allow exclusive evaluation of dentin–sealer adhesion. After sealer placement, AH Plus specimens were stored at 37 °C under relative humidity for 7 days. Bio-C Sealer specimens were stored at 37 °C and 100% relative humidity for 24 h. Subsequently, the specimens were maintained for an additional 6 days in hermetically sealed containers containing distilled water at the bottom, without direct contact between the liquid and the specimens, to provide a humid environment favorable for hydration of the calcium silicate-based sealer. These storage protocols were selected according to the setting mechanisms of each material, since Bio-C Sealer is a hydraulic calcium silicate-based sealer that requires moisture for continued hydration, whereas AH Plus is an epoxy resin-based sealer that sets independently of external water availability. The protocols followed the manufacturers’ instructions and were based on previous studies evaluating these sealers.

Tensile tes Although data normality ting was performed using a universal testing machine equipped with a custom-made device and a 100-N load cell. Tensile load was applied at a constant crosshead speed of 0.5 mm/min until failure. The maximum stress sustained by the dentin–sealer interface was recorded and expressed in MPa using the same formula described in the push-out test.

### Failure mode classification

After the push-out and tensile tests, failure modes were evaluated under a stereomicroscope under 20 × magnification (Leica Microsystems-M80, Wetzlar, Germany). Failures were classified as adhesive, when dentin surfaces were free of sealer; cohesive, when sealer remnants were present on dentin surfaces; or mixed, when both failure patterns were observed.

### Statistical analysis

Data normality for the push-out and tensile bond strength tests was assessed using the Shapiro–Wilk test, and homogeneity of variances was evaluated using Levene's test. As the data showed normal distribution and homoscedasticity, comparisons among groups were performed using one-way ANOVA, followed by Tukey's post hoc test. The significance level was set at α = 0.05. Although data normality was assessed using the Shapiro–Wilk test, the relatively small sample size per group may limit the sensitivity of this test to detect moderate deviations from normality. Therefore, the statistical findings should be interpreted considering this limitation.

SEM, EDS, and micro-FTIR analyses were performed descriptively to characterize dentin surface morphology, elemental composition, and interfacial chemical features. Failure mode distribution was expressed as percentages.

## Results

### SEM analysis

**Fig. 3:**
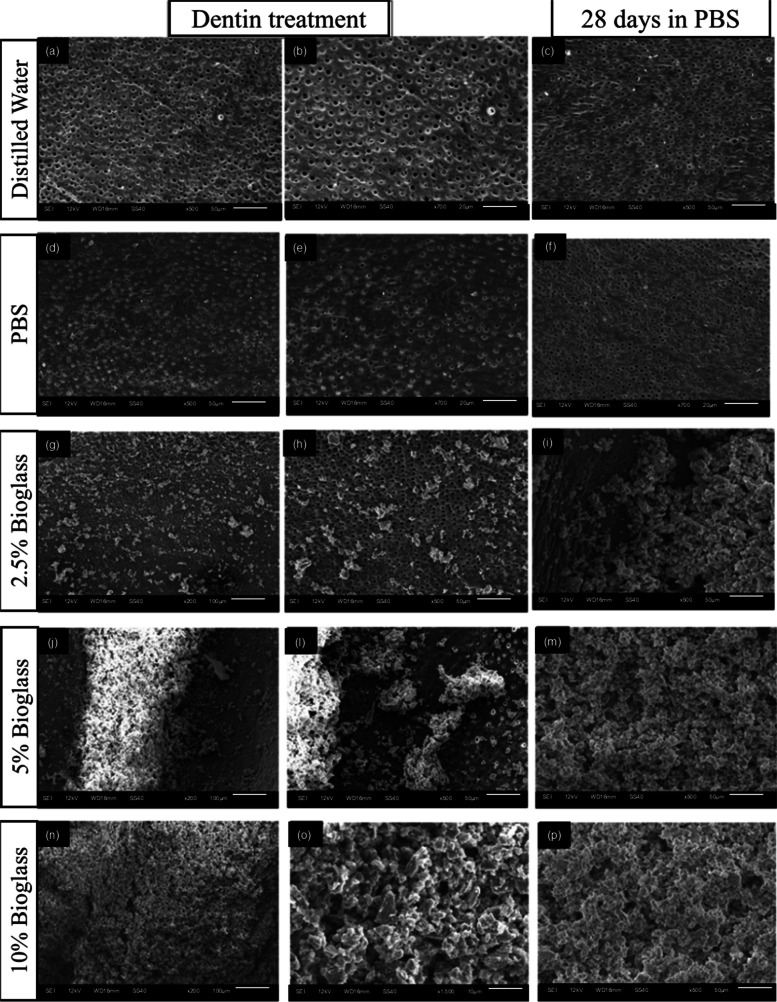
Representative scanning electron microscopy (SEM) images of root canal dentin surfaces after different surface treatments, acquired at the magnifications indicated by the scale bars. **a** and **b** Dentin treated with distilled water, showing predominantly open dentinal tubules (score 0); **c** same group after 28 days of PBS immersion (score 0). **d** and **e** Dentin treated with PBS, presenting partial tubule occlusion (score 1); **f** same group after 28 days of PBS immersion (score 1). **g** and **h** Dentin treated with 2.5% F18, showing partial surface coverage by deposited material (score 2); **i** same group after 28 days of PBS immersion, with increased deposition of bioactive material (score 3). **j** and** l** Dentin treated with 5% F18, exhibiting extensive surface coverage and marked dentinal tubule occlusion (score 4); **m** same group after 28 days of PBS immersion, with dense and continuous bioactive layer formation (score 5). **n** and **o** Dentin treated with 10% F18, showing extensive surface coverage immediately after treatment (score 4); **p** same group after 28 days of PBS immersion, with dense bioactive material deposition covering the dentin surface (score 5)

Representative SEM images are shown in Fig. 3. The distilled water group exhibited dentin surfaces with widely open dentinal tubules immediately after treatment, which remained largely evident after 28 days of PBS immersion (Fig. 3a–c) (score 0). In the PBS group, fine surface deposits and partial tubule occlusion were observed after treatment, with more pronounced surface deposition after 28 days of immersion, although several dentinal tubules remained visible (Fig. 3d–f) (score 1). Treatment with 2.5% F18 resulted in irregular particulate deposition and discontinuous surface coverage, with persistence of exposed dentinal tubules (Fig. 3g and h) (score 2). After 28 days of PBS immersion, a marked increase in surface deposits was observed, characterized by dense mineral agglomerates covering larger areas of the dentin surface (Fig. 3i) (score 3). In contrast, dentin treated with 5% and 10% F18 showed extensive surface coverage immediately after treatment, with limited exposure of dentinal tubules at higher magnification (Fig. 3j, l, n and o) (score 4). After 28 days of PBS immersion, both concentrations exhibited dense and more continuous surface deposits, suggesting a more stable mineral deposition pattern on the dentin surface (Fig. 3m and p) (score 5).

Although SEM images and scoring data suggest greater dentinal tubule occlusion and surface deposition after treatment with 5% and 10% F18, these findings should be interpreted as semi-quantitative morphological evidence supporting the surface changes observed.

### EDS analysis

EDS analysis demonstrated differences in the elemental composition of dentin surfaces according to the surface treatment and PBS immersion (Table 2). Specimens treated with distilled water or PBS showed lower calcium and phosphate contents compared with the F18-treated groups, although an increase in these elements was observed after 28 days of PBS immersion, indicating limited surface mineral deposition. In contrast, dentin treated with F18 suspensions exhibited higher levels of calcium and phosphate, which became more pronounced after PBS immersion. This effect was particularly evident in the 5% and 10% F18 groups, which presented greater calcium and phosphate contents compared with the control protocols and the lower F18 concentration. The presence of silicon and sodium exclusively in the F18-treated specimens further confirms the deposition of bioactive material on the dentin surface. Collectively, these findings indicate enhanced surface mineral deposition and support the bioactive behavior of F18-treated dentin following PBS immersion.

**Table 2:** Energy-dispersive X-ray spectroscopy (EDS) analysis showing the elemental composition (%) of dentin surfaces without PBS immersion (N/PBS) and after 28 days of PBS immersion (PBS 28 d) following different surface treatments

Groups	Si (%)		Na (%)		P (%)		Ca (%)	
	N/PBS	PBS 28 d	N/PBS	PBS 28 d	N/PBS	PBS 28 d	N/PBS	PBS 28 d
Distilled water	0.0	0.0	0.3	5.09	1.2	7.20	4.7	11.80
PBS	0.0	0.0	2.1	4.64	3.3	9.61	5.0	11.77
2.5% F18	9.6	13.98	0.7	7.44	2.5	15.08	8.7	21.20
5% F18	12.7	14.91	4.5	5.01	2.9	12.09	9.7	16.64
10% F18	17.2	16.70	4.7	4.13	1.2	12.30	11.2	23.80

N/PBS: analysis performed immediately after dentin surface treatment, without immersion in phosphate-buffered saline (PBS)

PBS 28 d: analysis performed after 28 days of immersion in PBS

2.5% F18, 5% F18, 10% F18: Bioglass F18 suspensions at the indicated concentrations

Si: silicon; Na: sodium; P: phosphorus; Ca: calcium

Mean values obtained from EDS analysis of three areas per specimen (*n =* 6), expressed as percentages (%)

### Micro-FTIR analysis

**Fig. 4:**
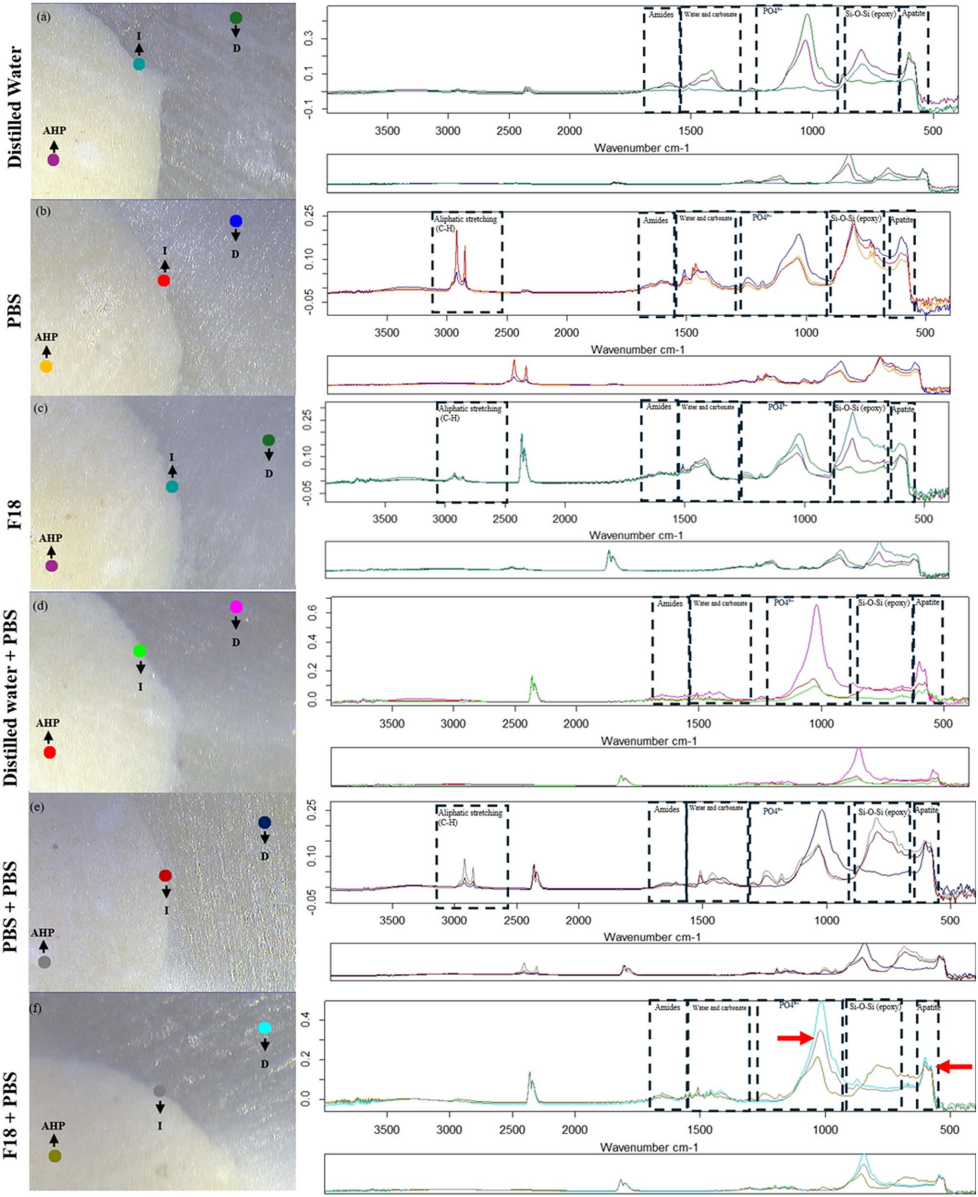
Representative micro–Fourier transform infrared (micro-FTIR) spectra obtained from AH Plus (AHP), the AHP/dentin interface (I), and dentin (D) after different dentin surface treatment protocols. **a** Distilled water; **b** PBS; **c** F18 treatment; **d** distilled water followed by 28 days of PBS immersion; **e** PBS followed by 28 days of PBS immersion; **f** F18 followed by 28 days of PBS immersion. The main absorption bands correspond to carbonate groups (~ 1410–1450 cm⁻^1^ and ~ 870 cm⁻^1^), phosphate groups (PO₄^3^⁻; ~ 960 cm⁻^1^ and 1000–1100 cm⁻^1^), water (H–O–H bending, ~ 1630 cm⁻^1^), and apatite-related bands, characterized by combined phosphate and carbonate signals below 600 cm⁻^1^ and around ~ 960 cm⁻.^1^. Increased intensity of phosphate- and apatite-related bands was observed mainly after PBS immersion, indicating enhanced mineral deposition at the sealer–dentin interface. This effect was more pronounced in the F18 + PBS group, as indicated by the red arrows. In all spectra, signals are presented from left to right as AHP, interface (I), and dentin (D)

**Fig. 5:**
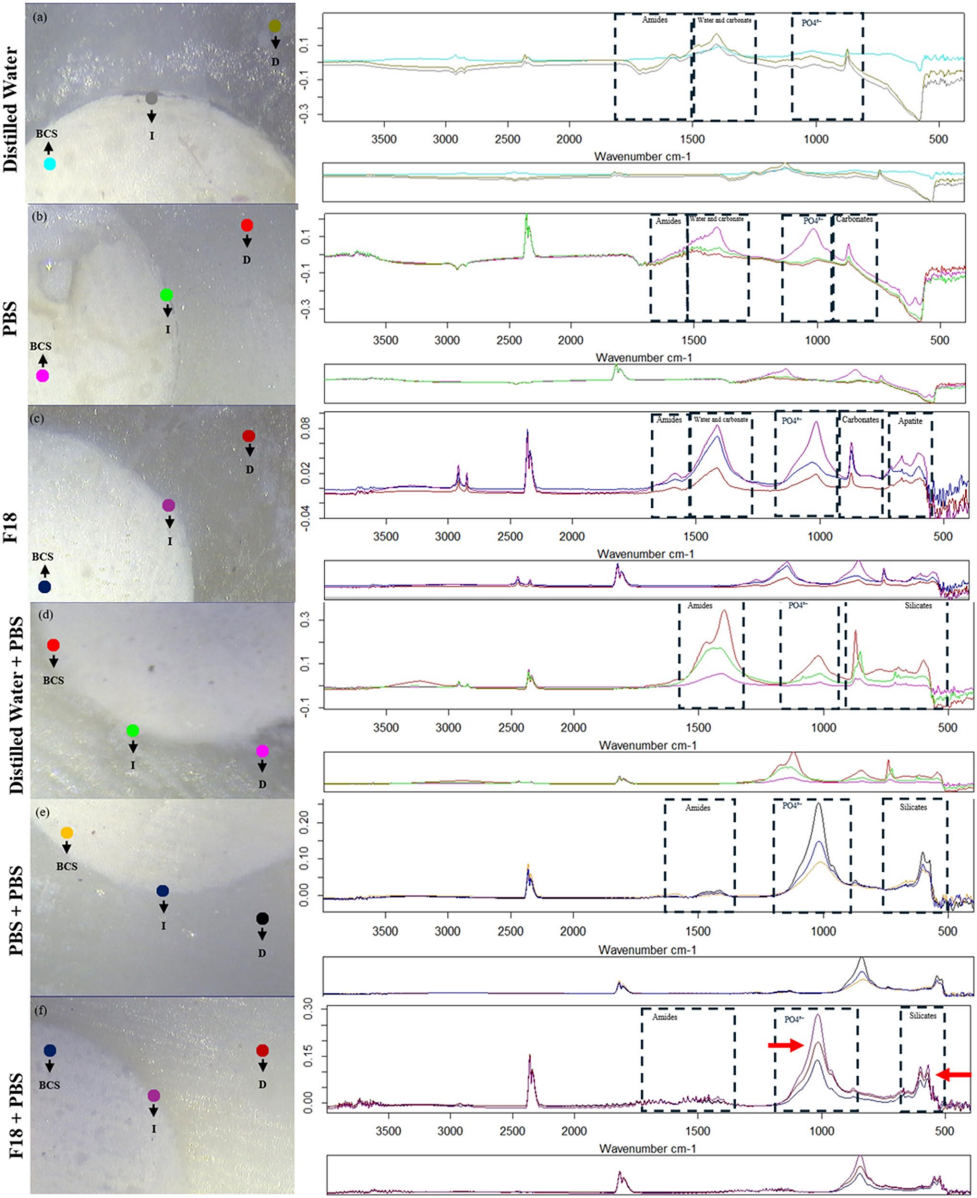
Representative micro–Fourier transform infrared (micro-FTIR) spectra obtained from Bio-C Sealer (BCS), the BCS/dentin interface (I), and dentin (D) after different dentin surface treatment protocols. **a** Distilled water; **b** PBS; **c** F18 treatment; **d** distilled water followed by 28 days of PBS immersion; **e** PBS followed by 28 days of PBS immersion; **f** F18 followed by 28 days of PBS immersion. The main absorption bands correspond to carbonate groups (~ 1410–1450 cm⁻^1^ and ~ 870 cm⁻^1^), phosphate groups (PO₄^3^⁻; ~ 960 cm⁻^1^ and 1000–1100 cm⁻^1^), water (H–O–H bending, ~ 1630 cm⁻^1^), and apatite-related bands, characterized by combined phosphate and carbonate signals below 600 cm⁻^1^ and around ~ 960 cm⁻.^1^. Increased intensity of phosphate- and apatite-related bands was observed mainly after PBS immersion, indicating enhanced mineral deposition at the sealer–dentin interface. This effect was more pronounced in the F18 + PBS group, as indicated by the red arrows. In all spectra, signals are presented from left to right as BCS, interface (I), and dentin (D)

Figures 4 and 5 show the micro-FTIR spectra obtained from dentin, the sealer–dentin interface, and the sealer after the different dentin surface treatment protocols. For AH Plus (Fig. 4)**,** the spectra demonstrated high chemical stability, with preservation of the characteristic absorption bands associated with the epoxy resin matrix under all experimental conditions. No relevant spectral changes were observed within the sealer itself or at the sealer–dentin interface immediately after surface treatment. However, at the dentin and at the sealer–dentin interface, immersion in PBS promoted a slight increase in the intensity of phosphate (~ 1030–1090 cm⁻^1^) and hydroxyl (~ 3570–3600 cm⁻^1^) bands, indicating initial mineral deposition on the dentin surface adjacent to the interface. Treatment with the experimental F18 suspension intensified these effects, with increased phosphate band intensity and the appearance of secondary carbonate-related peaks (~ 1410–1460 cm⁻^1^ and ~ 870 cm⁻^1^). At the sealer–dentin interface, concomitant increases in the bands at ~ 560 and ~ 600 cm⁻^1^, typically associated with phosphate vibrational modes of apatite-like phases, were observed, indicating enhanced interfacial mineral deposition. After 28 days of PBS immersion, these inorganic bands became more intense, suggesting progressive calcium phosphate–rich mineral accumulation at the sealer–dentin interface, particularly in the F18-treated groups.

For Bio-C Sealer (Fig. 5), the initial spectra exhibited intense phosphate (PO₄^3^⁻, ~ 1030–1090 cm⁻^1^ and ~ 560–600 cm⁻^1^) and silicate (Si–O–Si, ~ 900–1100 cm⁻^1^) bands, consistent with its calcium silicate–based composition. Dentin spectra showed preservation of the amide I (~ 1650 cm⁻^1^) and amide II (~ 1550 cm⁻^1^) bands associated with the collagen matrix, while only minor compositional changes were observed at the sealer–dentin interface in the absence of PBS immersion. Dentin treatment with PBS promoted increased phosphate, carbonate (~ 1410–1460 cm⁻^1^ and ~ 870 cm⁻^1^), and hydroxyl band intensities at the sealer–dentin interface, indicating activation of bioactive processes and the formation of an apatite-like calcium phosphate–rich mineral phase. These changes were markedly enhanced in the F18-treated group, with a pronounced increase in phosphate and carbonate bands and the appearance of peaks associated with hydrated calcium silicate, consistent with greater ionic release and secondary mineral phase formation. After 28 days of PBS immersion, intensified inorganic bands and attenuation of organic amide signals were observed at the sealer–dentin interface, indicating increased mineral deposition and partial masking of the underlying collagen matrix. These effects were more pronounced in the F18-treated specimens, confirming the high bioactive potential of F18 at the sealer–dentin interface.

### Push-out and tensile bond strength analysis

In the push-out test, dentin treatment with F18 significantly increased sealer–dentin bond strength at all tested concentrations (2.5%, 5%, and 10%) when compared with the control protocols (distilled water and PBS) (*p* < 0.05) (Table 3). Under the same surface treatment, AH Plus consistently exhibited higher bond strength than Bio-C Sealer (*p* < 0.05) (Table 3).

**Table 3:** Mean ± standard deviation of the push-out bond strength (MPa) of AH Plus (AHP) and Bio-C Sealer (BCS) after dentin treatment with distilled water, PBS, and F18 suspensions at different concentrations

	Distilled Water	PBS	2.5% F18	5% F18	10% F18
AHP	7.83 ± 1.47^aA^	6.87 ± 1.92^aA^	13.75 ± 4.07^bA^	13.85 ± 2.57^bA^	17.38 ± 4.78^bA^
BCS	2.87 ± 0.57^aB^	3.10 ± 0.63^bB^	5.23 ± 1.20^cB^	5.67 ± 0.44^cB^	5.45 ± 0.91^cB^

Lowercase letters in the same row indicate statistically significant differences among surface treatments (*p* < 0.05)

Uppercase letters in the same column indicate statistically significant differences between sealers (*p* < 0.05)

PBS: phosphate-buffered saline; F18: F18 bioactive glass; AHP: AH Plus; BCS: Bio-C Sealer

In the tensile test, dentin pre-treatment with 5% F18 resulted in the highest bond strength values for both sealers (*p* < 0.05) (Table 4). PBS significantly increased the bond strength of Bio-C Sealer compared with distilled water (*p* < 0.05), whereas AH Plus showed no significant difference between these two control protocols (*p* > 0.05). Nevertheless, both sealers exhibited significantly higher tensile bond strength after treatment with 5% F18 (*p* < 0.05) (Table 4).

**Table 4:** Mean ± standard deviation of the tensile bond strength (MPa) of AH Plus (AHP) and Bio-C Sealer (BCS) after dentin treatment with distilled water, PBS, and 5% F18

	Distilled Water	PBS	5% F18
AHP	1.32 ± 0.11^aA^	1.51 ± 0.07^aB^	2.23 ± 0.19^bA^
BCS	0.20 ± 0.02^aB^	0.41 ± 0.05^bB^	0.95 ± 0.18^cB^

Lowercase letters in the same row indicate statistical differences among treatments (*p* < 0.05)

Uppercase letters in the same column indicate statistical differences between sealers (*p* < 0.05)

PBS: phosphate-buffered saline; F18: Bioglass F18; AHP: AH Plus; BCS: Bio-C Sealer

### Failure mode analysis

In the push-out test, AH Plus showed a predominance of cohesive failures across all surface treatments (66–77%) (Table 5). Bio-C Sealer predominantly exhibited mixed failures in most groups. Adhesive failures were more frequent after PBS and particularly after 2.5% F18, whereas a near-balanced distribution between mixed and cohesive failures was observed after 10% F18 (Table 5). In the tensile test, adhesive failure predominated for both sealers under all conditions, reaching 100% adhesive failures after 5% F18 (Table 6).

**Table 5:** Frequency (%) of failure modes in the push-out bond strength test for AH Plus (AHP) and Bio-C Sealer (BCS)

	Failure Mode	Distilled Water	PBS	2.5% F18	5% F18	10% F18
	Mixed	25%	34%	50%	36%	23%
AHP	Adhesive	-	-	8%	-	-
	Cohesive	75%	66%	42%	64%	77%
	Mixed	42%	42%	51%	62%	48%
BCS	Adhesive	-	25%	49%	8%	2%
	Cohesive	58%	33%	-	30%	50%

Absence of failure mode (–). Phosphate-buffered saline (PBS). Bioglass F18 (F18). AH Plus (AHP). Bio-C Sealer (BCS)

**Table 6:** Frequency (%) of failure modes observed in the tensile bond strength test for AH Plus (AHP) and Bio-C Sealer (BCS)

	Failure Mode	Distilled Water	PBS	5% F18
	Mixed	25%	16%	-
AHP	Adhesive	75%	84%	100%
	Cohesive	-	-	-
	Mixed	42%	25%	-
BCS	Adhesive	58%	75%	100%
	Cohesive	-	-	-

Absence of failure mode (-). Phosphate-buffered saline (PBS). Bioglass F18 (F18). AH Plus (AHP). Bio-C Sealer (BCS)

## Discussion

The present study investigated whether dentin pre-treatment with a bioactive glass suspension could modify dentin surface characteristics and improve the interaction between root dentin and endodontic sealers. Surface bioactive potential and effective bonding at the dentin–sealer interface are essential for achieving a stable three-dimensional seal and long-term success of endodontic treatment [49], as previously demonstrated by studies on calcium silicate–based materials and bioactive glasses [50, 51]. The main finding of this study was that dentin treatment with F18 bioactive glass, particularly at the 5% concentration, promoted marked surface biomineralization and significantly increased the bond strength of both resin-based and bioceramic sealers. This effect was associated with extensive surface coverage by bioactive deposits and the formation of apatite at the dentin interface, indicating a favorable chemical interaction between the treated dentin surface and the sealing materials. Based on these findings, the null hypothesis was rejected, as dentin pre-treatment with F18 produced measurable changes in dentin surface characteristics and significantly enhanced bonding at the sealer–dentin interface. Our results are in agreement with studies that evaluated other dentin biomodification agents. Among these materials, experimental chitosan-based suspensions containing modified nano-hydroxyapatite (n-HA) promoted collagen fiber reinforcement, mineral deposition in the dentin structure, and inhibition of metalloproteinases [52]. Similarly, biomimetic mineral deposition approaches applied to eroded dentin, using calcium phosphate agents combined with plant-derived biomodifiers, increased mineral content and surface deposition in dentin [53]. Furthermore, gelatin-modified bioactive glass formulations demonstrated improved biocompatibility and more uniform and rapid remineralization of demineralized dentin [54]. These studies, along with the present findings, suggest that bioactive agents can improve dentin-material interactions.

In this investigation, SEM analysis demonstrated that dentin surface morphology was markedly influenced by the type of surface treatment applied. While distilled water and PBS resulted in dentin surfaces with predominantly open or only partially occluded dentinal tubules, treatment with F18 bioactive glass promoted pronounced surface modifications. At higher concentrations, particularly 5% and 10%, F18 induced extensive surface coverage and progressive deposition of mineralized material after immersion in PBS. The present study indicates that F18 promoted surface mineral deposition, leading to partial or complete dentinal tubule obliteration and the formation of a continuous mineral layer on dentin. Comparable surface changes have been reported for other bioactive glasses, which interact with aqueous media to release calcium and phosphate ions and subsequently promote apatite precipitation on mineralized substrates [13, 17, 18, 50]. Because the PBS solution was not renewed during the 28-day immersion period, changes in the ionic equilibrium may have occurred over time, including possible ion depletion and accumulation of reaction by-products. Thus, the mineral deposition observed in this study should be interpreted within the context of a static in vitro model, rather than as a direct simulation of the dynamic physiological environment.

It should be noted that the present study employed a static PBS immersion model without solution renewal over the 28-day period. Although this approach allows for standardized long-term evaluation of mineral deposition, it may also lead to ion accumulation within the medium, resulting in localized supersaturation conditions. Under such circumstances, precipitation of calcium phosphate phases may occur not only as a consequence of the intrinsic bioactivity of Bioglass F18, but also due to physicochemical processes driven by ionic saturation of the surrounding environment. Therefore, while the observed elemental increases and FTIR-detected mineral phases are consistent with biomineralization, they may also reflect a combined effect of material-induced bioactivity and solution-mediated precipitation phenomena. This limitation should be considered when interpreting the extent of the bioactive response under static in vitro conditions.

The SEM/EDS findings should be interpreted with caution because the same dentin regions were not evaluated before and after PBS immersion. This qualitative, non-site-specific approach may introduce variability related to local differences in dentin surface morphology and limits the interpretation of localized changes over time. Therefore, these analyses should be considered descriptive evidence of overall surface and elemental deposition patterns. Future studies should incorporate standardized region tracking, fiducial markers, or other reproducible localization methods to improve the reliability of longitudinal SEM/EDS evaluations.

The elemental findings obtained by EDS supported and complemented the morphological observations from SEM. In this study, dentin surfaces treated with F18 exhibited higher calcium and phosphate levels than the control groups, an effect that became more pronounced after 28 days of PBS immersion. The exclusive presence of silicon in the F18-treated specimens further confirms the deposition of glass-derived material on the dentin surface. Taken together, these compositional changes indicate an active ion exchange process and the formation of calcium- and phosphate-rich deposits, which are hallmarks of bioactive glass behavior. Consistent with previous investigations, the release of calcium, phosphate, and silicon ions plays a central role in apatite-like mineral formation and surface bioactive potential, thereby reinforcing the interpretation of the present findings [13, 14, 17, 18, 55].

Micro-FTIR analysis demonstrated that dentin pre-treatment with F18 markedly affected the chemical characteristics of the Bio-C Sealer–dentin interface, particularly after 28 days of PBS immersion. In the present study, the spectra obtained from the interface showed intensified phosphate and carbonate bands, which are consistent with the formation of a carbonated apatite-like calcium phosphate phase, a typical indicator of mineral deposition associated with bioactive processes. These changes were more pronounced in the F18-treated groups, supporting the occurrence of an active bioactive potential process at the sealer–dentin interface. Similar spectral patterns associated with apatite-like calcium phosphate precipitation and mineral layer maturation have been widely reported for bioactive glasses and calcium silicate–based materials exposed to phosphate-containing suspensions [13, 17, 18, 51, 56]. Therefore, the present findings indicate that F18 enhances interfacial mineral deposition when associated with a bioceramic sealer.

For AH Plus, the micro-FTIR spectra revealed high chemical stability of the epoxy resin matrix, with preservation of its characteristic absorption bands under all experimental conditions. In the present micro-FTIR evaluation, only discrete spectral changes were detected in dentin and at the sealer–dentin interface after PBS immersion, including a slight increase in phosphate-related bands and a modest reduction in organic signals. These findings suggest limited interfacial mineral deposition rather than degradation of the resin sealer itself. Previous studies have shown that resin-based sealers do not exhibit intrinsic bioactivity but may benefit from mineralized substrates, which can improve micromechanical interlocking and interfacial adaptation [10, 11]. Thus, although AH Plus does not actively induce mineral formation, the presence of a mineralized dentin surface promoted by F18 may favor interfacial interactions.

Micro-FTIR findings obtained in this investigation provide a chemical basis for the increase in bond strength observed in the mechanical tests. The formation of a mineralized layer at the sealer–dentin interface, evidenced by intensified phosphate and carbonate bands, supports the occurrence of hydroxycarbonate apatite deposition, which plays a key role in strengthening interfacial interaction [50, 51]. This effect was particularly evident for Bio-C Sealer, whose bioactive behavior favors chemical bonding and interfacial stability when in contact with a mineralized substrate [57]. In the present experimental design, bond strength was assessed using endodontic sealers alone, thereby minimizing the influence of gutta-percha deformation and enabling a more direct evaluation of adhesion to dentin [57, 58]. Accordingly, although extensive surface coverage was observed at the 5% and 10% F18 concentrations, the mechanical outcomes reflect exclusively the dentin–sealer interaction. Future investigations incorporating gutta-percha protocols may further clarify whether F18-induced surface mineralization influences overall material adaptation under clinical condition. Although the present findings demonstrate a consistent association between surface biomineralization and increased bond strength, it cannot be assumed that mineral deposition alone accounts for the observed mechanical behavior. Other factors, such as physicochemical changes in dentin induced by the treatment environment, may also contribute. Therefore, future studies including additional controls are warranted to better elucidate these mechanisms.

Dentin treatment with F18 increased bond strength in the push-out test for both Bio-C Sealer and AH Plus at all tested concentrations. This effect can be explained by the distinct adhesion mechanisms of each sealer. Calcium silicate–based sealers undergo hydration reactions that result in the formation of calcium silicate hydrate and calcium hydroxide, which favor ionic exchange and interfacial mineral deposition [59]. In contrast, the adhesion of the epoxy resin–based sealer AH Plus relies primarily on covalent interactions between epoxy groups and collagen fibers within root dentin [60]. Although collagen-mediated adhesion is a key mechanism for epoxy resin-based sealers [61] the present findings suggest that mineral-modified dentin surfaces may provide an additional substrate for micromechanical interaction, rather than acting as a competing mechanism. In this context, the higher bond strength observed for AH Plus may be related to the formation of apatite-like calcium phosphate precipitates at the dentin surface following F18 treatment, which likely enhanced micromechanical interlocking and tag formation at the sealer–dentin interface [11, 62]. Although AH Plus typically exhibits higher bond strength than Bio-C Sealer, as consistently reported in the literature [37, 58, 60], the dentin pre-treatment protocol proposed in this investigation reduced the magnitude of this difference, indicating that F18-induced biomineralization positively modulated the adhesion of both root canal sealer types.

The absence of rinsing after F18 treatment should be considered when interpreting the bond strength results. Although this approach was used to preserve the bioactive particles deposited on the dentin surface, residual particles or solution components may have remained at the interface and could have influenced sealer adaptation, interfacial characteristics, and bond strength. Future studies may compare rinsed and non-rinsed protocols to determine how post-treatment handling affects the stability and performance of the F18-modified dentin interface.

In the experimental conditions evaluated in this investigation, failure mode analysis provided clinically relevant information regarding the quality and stability of the sealer–dentin interface. In the push-out test, AH Plus predominantly exhibited cohesive failures, indicating that bond strength exceeded the internal strength of the material and suggesting a mechanically stable interface. For Bio-C Sealer, dentin pre-treatment with F18 modified the failure pattern, with a higher frequency of mixed failures at the 5% concentration, reflecting a more integrated interface likely reinforced by surface mineral deposition. This concentration-dependent behavior suggests that F18 promotes interfacial maturation without inducing brittle adhesion, a feature that is clinically desirable because it favors stress distribution at the sealer–dentin interface rather than abrupt interfacial failure [63–65]. In the tensile test, adhesive failures predominated for both sealers, including the F18-treated groups. Rather than indicating ineffective bonding, this pattern highlights the ability of tensile loading to reveal localized interfacial discontinuities under axial stress conditions. Similar failure distributions have been reported even in systems showing improved interfacial interaction and biomineralization [63–65]. From a clinical perspective, the combined interpretation of failure modes obtained from push-out and tensile tests supports the notion that F18 enhances interfacial quality while maintaining mechanical compatibility under different loading scenarios relevant to endodontic function.

Nevertheless, the predominance of adhesive failures observed in the F18-treated groups warrants caution. As the specimens were not rinsed after F18 application, residual bioactive deposits may have induced the formation of a modified interfacial layer with distinct mechanical properties from those of both dentin and the sealer. Consequently, it cannot be ruled out that these adhesive failures occurred within or adjacent to this modified zone. Further ultrastructural investigations are required to map the precise locus of failure and clarify how F18-induced surface alterations modulate the interfacial fracture mechanics.

From a mechanical standpoint, push-out and tensile tests are complementary methodologies for evaluating the bond strength of endodontic sealers, as they apply distinct loading vectors and therefore probe different aspects of the sealer–dentin interface [66, 67]. The push-out test applies a load perpendicular to the interface and is particularly sensitive to shear resistance and to the quality of the sealer’s lateral adaptation to dentin walls [68]. In contrast, the tensile test imposes an axial load, which may reveal interfacial weaknesses that are not necessarily detected under shear-dominated conditions [69, 70]. Accordingly, satisfactory performance in push-out testing does not always translate into equivalent resistance under tensile loading. Axial resistance is more dependent on the internal cohesion of the material and its penetration into dentinal tubules, which contribute to mechanical retention, whereas push-out resistance is more strongly influenced by the integrity and continuity of the sealer–dentin interface [70, 71]. The combined use of these tests therefore provides a broader and more balanced assessment of interfacial behavior and mechanical stability, with lateral adhesion being predominantly captured by push-out testing and axial retention more effectively evaluated by tensile testing.

The different storage protocols used for Bio-C Sealer and AH Plus should be considered when interpreting the tensile bond strength results. These conditions were intentionally selected according to the distinct setting mechanisms of the materials. Bio-C Sealer is a hydraulic calcium silicate–based material that requires moisture availability to complete its hydration reaction, whereas AH Plus undergoes a resin-based polymerization process that does not depend on external moisture. To provide favorable conditions for hydration, Bio-C Sealer specimens were stored in hermetically sealed containers containing distilled water at the bottom of the vial, without direct contact between the liquid and the specimens, thereby creating a humid environment. In contrast, AH Plus specimens were maintained under conditions appropriate for their resin-based setting reaction. Therefore, the objective was not to expose both materials to identical storage conditions, but rather to allow adequate maturation according to their physicochemical characteristics. Nevertheless, because differences in storage conditions may influence material maturation and bond strength development, direct comparisons between the absolute tensile bond strength values of Bio-C Sealer and AH Plus should be interpreted with caution. Accordingly, the tensile bond strength results are more appropriately interpreted as the effect of dentin pretreatment within each sealer than as a direct comparison between the two materials.

The analytical methods used in this study, including SEM, EDS, micro-FTIR, and mechanical testing, are well-established in dental materials research [57, 72, 73]. Their combined application allowed a complementary assessment of the dentin–sealer interface after F18 bioactive glass pretreatment. The present findings suggest that F18 may modify dentin surface characteristics, promote mineral deposition, and influence early sealer–dentin bond strength. Therefore, the contribution of this study lies in the integrated evaluation of surface, chemical, and mechanical outcomes related to F18-treated dentin, rather than in the introduction of a new analytical method.

Although the present findings demonstrate that dentin pre-treatment with F18 bioactive glass positively influenced interfacial characteristics and bond strength, some methodological limitations should be acknowledged. This study did not evaluate the ultrastructural pattern of mineral deposition nor the mechanical recovery of dentin through nanoindentation analysis. Therefore, although mineral deposition was observed on the dentin surface, it was not possible to determine whether this process occurred in the intrafibrillar compartment or resulted in functional mechanical restoration. Consequently, the findings should be interpreted as evidence of superficial mineral deposition and bioactive potential, and not as definitive confirmation of intrafibrillar remineralization. In addition, the micro-FTIR analysis was performed as a qualitative characterization method and did not include quantitative spectroscopic indices, such as mineral-to-matrix or carbonate-to-phosphate ratios. Therefore, the spectroscopic findings should be interpreted as complementary evidence of interfacial chemical changes rather than as a quantitative assessment of mineralization. Furthermore, this investigation was conducted under controlled laboratory conditions and evaluated a limited number of sealers and experimental variables, which may not fully reproduce the biological and mechanical challenges present in vivo. Another methodological aspect was the absence of pH measurement during the application of F18, which could further elucidate its bioactive mechanism. Bovine teeth were used for micro-FTIR and mechanical tests to promote greater standardization of the samples. Although bovine dentin presents similarities in elemental composition [46] and is considered a suitable substrate in relation to human dentin [47], without significantly influencing the bond strength of root canal obturation [47], structural differences may influence the results [48]. In addition, although the dentin specimens were standardized through controlled root length, canal preparation, and slice thickness, direct morphometric matching of root dimensions and dentin thickness was not performed. Therefore, residual anatomical variability may have influenced the mechanical outcomes. Accordingly, the results should be interpreted considering these experimental limitations.

A limitation of the present study is that the same specimens were not evaluated both before and after PBS immersion, which may limit direct comparisons. In addition, dentin specimens were longitudinally sectioned prior to surface treatment, which does not fully reproduce the fluid dynamics and confinement conditions of irrigants within the root canal system. Consequently, the SEM/EDS findings should be interpreted as an assessment of the bioactive potential of F18 under controlled laboratory conditions rather than as a direct simulation of clinical irrigant–dentin interactions. Furthermore, since different regions and specimens were analyzed before and after treatment, SEM/EDS observations should be considered complementary descriptive evidence of surface modification. However, multiple standardized regions per specimen were analyzed to provide a representative assessment of surface morphology. Moreover, interfacial mineral deposition can influence failure patterns differently under shear versus axial tension. From a clinical standpoint, the additional 3-min pretreatment step with F18 may increase clinical treatment time; however, its duration is comparable to conventional irrigation procedures routinely performed in endodontic practice. Nevertheless, future strategies could incorporate F18 directly into endodontic obturation sealer formulations, optimizing clinical practicality. Therefore, future studies incorporating intact specimens and closed canal systems, as well as aging protocols, cyclic loading, and in vivo models, are needed to better reproduce clinical conditions and to clarify the mechanical stability of the mineralized layer and the long-term behavior of the dentin interface treated with F18.

The short exposure time and limited solution volume used in this study should be considered when interpreting the dentin surface modification and bond strength results. These parameters may influence the extent and reproducibility of chemical and structural changes on dentin surfaces and limit direct extrapolation to clinical irrigation conditions [74, 75]. Although the SEM, EDS, and mechanical findings suggest treatment-related effects, future studies should evaluate different exposure times and solution volumes to determine whether these effects are maintained under protocols that more closely resemble clinical irrigation procedures.

The mechanical results presented in this study represent short-term outcomes and should be interpreted with caution when extrapolating to clinical performance. Root canal fillings are subjected to complex conditions over time, including thermal fluctuations, mechanical loading, and continuous exposure to fluids, which may affect the durability of the sealer–dentin interface. Future studies incorporating aging protocols, such as thermocycling, mechanical fatigue, and long-term storage, are necessary to evaluate the long-term stability of the mineralized interface induced by F18. Although the statistical analysis allowed comparisons among treatments within each sealer and between sealers within the same treatment condition, the study was not analyzed using a factorial model. Therefore, potential interaction effects between surface treatment and sealer type were not formally evaluated. In addition, push-out and tensile bond strength were analyzed as independent mechanical outcomes without adjustment for multiple testing across endpoints. Therefore, the findings should be interpreted considering the potential increase in the overall probability of Type I error.

Overall, the findings of this investigation indicate that dentin surface modification with a bioactive glass suspension represents a promising adjunctive strategy to enhance the interaction between root dentin and endodontic sealers. By promoting surface biomineralization and improving interfacial bonding without compromising mechanical compatibility, F18 pre-treatment may contribute to a more stable and durable sealing of the root canal system, thereby supporting long-term endodontic success.

## Conclusion

Dentin pre-treatment with Bioglass F18 promoted surface biomineralization and improved the sealer–dentin interfacial characteristics by inducing mineral deposition and the formation of a formation of a modified interfacial layer. All tested concentrations (2.5%, 5%, and 10%) improved sealer–dentin bond strength compared with control conditions. In push-out testing, no statistically significant differences were observed between 5 and 10% F18. In tensile testing, 5% F18 improved bond strength compared with the control condition. Overall, Bioglass F18 represents a promising adjunctive strategy to optimize dentin bioactivity and improve the mechanical performance of root canal sealers. Within the limitations of this short-term in vitro study, 5% F18 showed favorable performance across key outcomes but without consistent superiority across all mechanical tests.

## Data Availability

No datasets were generated or analysed during the current study.
